# Bioresorbable scaffolds: everything resolved?

**DOI:** 10.1007/s12471-015-0650-4

**Published:** 2015-01-28

**Authors:** Yolande Appelman

**Affiliations:** VU University Medical Center, Amsterdam, The Netherlands

Coronary artery disease (CAD) is still the leading cause of death in the Western world, resulting in 17.5 million deaths in 2012. Treatment and research have focused on primary and secondary prevention and on improvement of percutaneous coronary intervention (PCI) techniques. The introduction of balloon angioplasty (1977) and later the bare metal stent (1994) gave an enormous reduction in the re-stenosis rate from 50–60 % to nearly 15–30 %. With the coming of the drug-eluting stent (DES) (2000) the in-stent restenosis rate has dropped to the current 4–10 %. Next to improvement of the stent device a change in medical treatment before and after stenting was implemented. Besides acetylsalicylic acid, treatment with a second antiplatelet drug was introduced to lower the in-stent restenosis rate and subsequent target lesion revascularisation (TLR) and to prevent in-stent thrombosis. Currently, TLR with DES is 3–7 % and in-stent thrombosis (including acute, early and late) around 1–2 % [1]. Although the introduction of DES was a major step forward, there is still concern regarding the vessel wall response. Issues that remain to be improved include in-stent restenosis, delayed endothelial healing, prolonged exposure of stent struts and the occurrence of (late) stent thrombosis. Furthermore, vessel caging results in impaired endothelial function, interferes with non-invasive imaging techniques leading to artefacts (CT and MRI) and possible impairment of future treatment options (re-PCI and CABG). Also the desired duration of dual antiplatelet therapy is unclear and still under investigation.

So, knowing the good results with DES the question is if there is still room and need for further improvement? After the introduction of the bioresorbable scaffold (BRS) and the promising results of the Absorb A and B trial, one would think that we are really missing the boat if we do not use this novel but still expensive device. The major advantage of the new scaffolds promises to overcome the aforementioned concerns with respect to the use of DES and seems to resolve them. Furthermore instead of vessel recoil late lumen enlargement has been observed. In this issue of the Netherlands Heart Journal, the article by Felix et al. nicely describes the results of the currently available BRS data [2]. Although research has been performed with different scaffolds, at this moment two are commercially available with the Conformité Européenne (CE) mark for use in CAD: the Absorb Bioresorbable Vascular Scaffold (BVS, Abbott Vascular) and the DESolve scaffold (Elixer Medical Corporation). The scaffolds are made of poly-L-lactic acid (PLLA) and elute a 1:1 mixture of PLLA and the antiproliferative drug everolimus. Full hydrolytic degradation takes as long as 3 years in patients. As most studies are performed with the BVS from Abbott, the results mentioned in this editorial refer to this scaffold.

The results of the paper by Felix et al. and of the recently published paper by Wiebe et al. demonstrate that there are still some questions to be answered and some critical comments to be made before justifying a widespread use of BVS [2, 3] Although the scaffold is resolved after 3 years, the stent struts are thick (150 µm), the device is still bulky, not very flexible and subsequently difficult to place in calcified lesions, distal small arteries and tortuous lesions. As pre-dilatation of the segment is mandatory, post-dilatations are mostly needed which leads to prolonged duration of the procedure, higher radiation doses and higher costs. Also, preferably the BVS should not be placed in bifurcation lesions as side branches are difficult to enter and ending the procedure with a final kissing balloon is not recommended because the stent struts will dislocate or break. Furthermore, the majority of the published data are not derived from randomised clinical trials (RCT), but are drawn from registries where imputation of data has been used or cohorts are compared with existing DES data. Only the Absorb II trial, which is still ongoing, is a RCT. Also, there is some concern with respect to late stent thrombosis, possibly due to the fact that it takes almost 3 years for the BVS to vanish completely. The ideal timing for absorption of the scaffold remains unclear. It is said that one of the advantages of the BVS is the restoration of vascular motion. The exact definition of vascular motion remains debatable as well as the way it should be measured. In addition, the clinical value of vasomotion as an outcome parameter needs to be established. There are several factors influencing vascular motion including risk factors (i.e. smoking, hypertension, diabetes), gender and stress among other factors [4]. In all the BVS studies vasomotion has been measured by the application of acetylcholine or methylergonovine assessing the response by angiography. The question is if this is the right way to measure vasomotion as circumstances between patients differ and neither cold-pressure test nor metabolic measurements are applied [5]. The answer might come from the ongoing VANISH trial, a single-centre RCT performed in the VU University medical center in Amsterdam in which PET scan and cold-pressure tests will be used to determine the (clinical) impact of vascular restoration with BVS in comparison with DES on endothelium-dependent vasodilation and maximal hyperaemic myocardial perfusion using H2 150 PET after 3 years of follow-up.

And how big is the problem for patients who need to undergo CABG after PCI with DES? The latest results with respect to TLR after DES are very good. In the Compare trial the Xience V results demonstrate any target vessel revascularisation of 3.2 %, re-PCI 2.5 % and CABG 0.8 %; any TLR of 2.9 %, re-PCI 2.1 % and CABG 0.8 % [6]. Consequently, the aforementioned advantage to more readily perform CABG after BVS is only envisaged for a very small group of patients.

In conclusion, the so-called next-generation ‘stent’ seems very promising and potentially could present a big step forward in interventional cardiology. However, further research in randomised clinical trials is needed, including cost-benefit analysis and during a long-term follow-up period to find out if BVS resolves the shortcomings of DES.

## Funding

None.

## Conflict of interests

None declared.

## References

[1] Sarno G, Lagerqvist B, Fröbert O (2012). Lower risk of stent thrombosis and restenosis with unrestricted use of ‘new-generation’ drug-eluting stents: a report from the nationwide Swedish Coronary Angiography and Angioplasty Registry (SCAAR). Eur Heart J.

[2] Felix C, Everaert B, Diletti R, et al. Current status of clinically available bioresorbable scaffolds in percutaneous coronary interventions. Neth Heart J. 2014. doi:10.1007/s12471-015-0652-2. 10.1007/s12471-015-0652-2 PMC4352158 25626697

[3] Wiebe J, Nef H, Hamm C. (2014). Current Status of Bioresorbable Scaffolds in the Treatment of Coronary Artery Disease. J Am Coll Cardiol.

[4] Maas AH, Appelman YE (2010). Gender differences in coronary heart disease. Neth Heart J.

[5] Schindler T, Nitzsche E, Olschewski M (2004). PET-measured responses of MBF to cold pressor testing correlate with indices of coronary vasomotion on quantitative coronary angiography. J Nucl Med.

[6] Smits P, Kedhi E, Royaards KJ (2011). 2-year follow-up of a randomized controlled trial of everolimus- and paclitaxel-eluting stents for coronary revascularization in daily practice. COMPARE (Comparison of the everolimus eluting XIENCE-V stent with the paclitaxel eluting TAXUS LIBERTÉ stent in all-comers: a randomized open label trial). J Am Coll Cardiol.

